# Economic consequences of ill-health for households in northern rural India

**DOI:** 10.1186/s12913-015-0833-0

**Published:** 2015-04-26

**Authors:** Marta Quintussi, Ellen Van de Poel, Pradeep Panda, Frans Rutten

**Affiliations:** Cologne Graduate School in Management, Economics and Social Sciences, University of Cologne, Cologne, Germany; Institute of Health Policy and Management, Erasmus University Rotterdam, Rotterdam, The Netherlands; Micro Insurance Academy, New Delhi, India

**Keywords:** Health shocks, Health expenditures, Coping strategies, Rural India

## Abstract

**Background:**

As compared to other countries in South East Asia, India’s health care system is characterized by very high out of pocket payments, and consequently low financial protection and access to care. This paper describes the relative importance of ill-health compared to other adverse events, the conduits through which ill-health affects household welfare and the coping strategies used to finance these expenses.

**Methods:**

Cross-sectional data are used from a survey conducted with 5241 households in Uttar Pradesh and Bihar in 2010 that included a household shocks module and detailed information about health care use and spending.

**Results:**

Health-related adverse events were the second most common adverse events (34%), after natural disasters (51%). Crop and livestock disease and weddings each affected about 8% of households. Only a fourth of households reported to have recovered from illness and/or death in the family (by the time of the survey). Most of the households’ economic burden related to ill-health was depending on direct medical costs, but indirect costs (such as lost earnings and transportation or food costs) were also not negligible. Close to half of the health expenditures were made for chronic conditions. Households tried to cope with health-related expenditures mostly by dissaving, borrowing and selling assets. Few households reported having to reduce (food) consumption in response to ill-health.

**Conclusions:**

In the absence of pre-financing schemes, ill-health events pose a substantial threat to household welfare in rural India. While most households seem to be able to smooth consumption in the short term, coping strategies like selling assets and borrowing from moneylenders are likely to have severe long term consequences. As most of the households’ economic risk related to ill-health appears to depend on out of pocket spending, introducing health insurance may contribute significantly to alleviate economic hardship for families in rural India. The importance of care for chronic diseases, however, represents a big challenge for the sustainability of community based health insurance schemes, since it is necessary to ensure a sufficient degree of risk pooling.

**Electronic supplementary material:**

The online version of this article (doi:10.1186/s12913-015-0833-0) contains supplementary material, which is available to authorized users.

## Background

In India, as in many developing countries, the bulk of health care expenditures is financed through out of pocket payments (OOP) made at the point of use [1]. In the absence of pre-financing mechanisms such as health insurance households confronted with ill-health are exposed to catastrophic expenditures or decide to forego essential medical treatment altogether. Illness is found to be one of the main reasons for falling into poverty in India [2,3]. Ill-health can have economic implications through multiple channels. Health care use involves both direct costs for doctor fees, tests and drugs, but also indirect costs, including transportation and foregone earnings for patients and their family members. While the latter costs are often not explicitly investigated, they have been shown to be not negligible [4-7]. Households without formal insurance often resort to alternative coping strategies, such as borrowing and selling assets, to finance health-related expenditures [8-13]. While health payments financed through these strategies are not at the expense of current consumption, they do entail long-term sacrifices.

While some papers have documented the degree of catastrophic spending and impoverishment related to ill-health [3,8,11,12], there is – to the best of our knowledge – no evidence on the relative importance of ill-health as compared to other adverse events^a^ and very limited evidence on the conduits through which ill-health affects households’ living standards in India [7,11,12]. This paper adds to the existing literature by comparing health shocks with other adverse events in terms of prevalence, cost, severity and recovery in rural India. Furthermore, this study aims at shedding light on the way ill-health affects households’ welfare in the target communities, by identifying the types of health-related expenditures that place the largest economic burden on households and by analysing the strategies households employ to finance these expenditures and their long term consequences. As most of the households’ economic risk deriving from ill-health appears to be related to OOP spending, authors make policy recommendations for alleviating economic hardship of families in rural India.

## Methods

The data derive from a randomized controlled trial of Community-based Health Insurance (CBHI) in three sites in rural India, precisely Kanpur Dehat and Pratapgarh districts in Uttar Pradesh, and Vaishali district in Bihar^b^. The CBHI schemes are targeted at Self-Help Groups (SHGs), which form a well-established informal micro-credit system throughout most of India [14]. A SHG typically consists of 12–15 women who pool resources and jointly decide on loans^c^. Baseline household data have been collected in 2010 for 5214 households (1751, 1541 and 1922 households in Kanpur Dehat, Pratapgarh and Vaishali respectively), representing 29880 individuals^d^. Data were collected from the entire population of households affiliated with SHGs (through at least one member), and from a random sample of the non-SHG population in each of the three sites. Sample weights have been constructed to adjust for the oversampling of SHG-related households. In accordance with the guidelines issued by the Indian Council of Medical Research in 2006 [15]^e^, the overall study and the English versions of all employed data collection tools were checked and approved by the Ethics Committee of the University of Cologne (Germany).

The survey contains a retrospective household shocks module, which asks households about different kinds of ‘adverse events’ that they have been confronted with in the year preceding the survey (natural disaster, storage/crop/livestock disease, job loss, drop in sale price of agricultural products, increase in agricultural input price, conflict, wedding, illness or death), how these affected them and how they coped with them (see Additional file 1). Notwithstanding that such retrospective tools can suffer from reporting bias, they provide useful information on the relevance and consequences of various threats to household welfare in the absence of panel data. However, such tools have not often been used in this context [16]. As some of the reported threats, such as weddings, obviously do not come unexpectedly, we prefer referring to adverse events as opposed to ‘shocks’ in the remainder.

Since the baseline data were also used as input for the calculation of insurance premiums (for the CBHI scheme), they contain many details on ill-health conditions, health care seeking behavior, costs and financing of health care^f^. For each illness episode (or pregnancy) of each household member (30 days recall for outpatient care and 12 months recall for inpatient care), we know symptoms, volume, location and detailed costs of health care use and financing mechanisms. Annual hospitalization costs have been divided by 12 to be comparable to other monthly health expenditures. Health care spending can be categorized along two dimensions: (i) the type of care (outpatient for chronic/acute conditions, inpatient care and maternity care), and (ii) the type of expenditures (fees, additional costs for drugs and tests, indirect costs related to travel and food of the patient and accompanying persons and productivity loss of the patient and/or accompanying persons). It should be noted that chronic conditions in this context relate to conditions that are reported to have been ongoing for 30 days or more, and can therefore also include more acute conditions that are not appropriately treated and therefore persistent. Furthermore, costs of chronic diseases are likely to be underestimated, since we only possess in-depth cost information for the last visit, while 31% of respondents reported to have received medical help more than once during the last month.

We have also tried to investigate heterogeneity of results across the type of ill-health condition (communicable versus non-communicable), using a classification based on symptoms (obtained through the ICD10 codes developed by the World Health Organization [17]). Detailed results are available upon request.

Regarding household characteristics, we construct variables related to demographics (the proportion of elderly over 65 years old, of children under the age of 13 and of women between 13 and 49 years old), indicator variables for SHG membership, scheduled caste/tribe status, Hindu religion and location. We hypothesize that, next to economic characteristics, social characteristics, such as scheduled caste/tribe status and religion, are important cultural indicators in these contexts and can influence the way ill-health events are correlated with households’ economic status. Desai and Dubey [18] show how caste affiliation determines households’ economic situation, community participation and access to education and healthcare. Several other studies also refer to caste status and/or religion, next to welfare status, as factors influencing health care access and financing [10-12,19].

Household socioeconomic status (SES) is measured by a principal component score, obtained from the analysis of asset ownership and household dwelling characteristics [20], which is used to divide the population in wealth thirds. We prefer this to household spending data, as it is less likely to be affected by ill-health and consumption of health care. As households reported to mostly sell agricultural items to finance health expenditures, we have excluded these items from the principal component analysis. Socioeconomic inequalities (in incidence of household shocks) are measured by a corrected concentration index (CI) for binary outcomes, as suggested by Erreygers [21]^g^. A CI is derived from a concentration curve which plots cumulative shares of the variable of interest ‘y’ against cumulative shares of the population ranked by socioeconomic status. The CI lies between −1 and +1, with greater values indicating greater SES inequality. Positive values indicate that ‘y’ is more concentrated among the wealthier households and vice versa.

Probit models are used to investigate determinants of coping strategies and the choice of moneylender among those households that borrow in response to ill-health.

## Results

### Household shocks module

The communities in the three different sites appeared quite homogeneous concerning most of the socio-economic characteristics and the prevalence and distribution of ill-health events (Table 1). The majority of households were of Hindu religion and belonged to scheduled castes/tribes or other backward castes. Average per capita expenditures was higher in Kanpur Dehat, but a larger share of households fell in the upper wealth quintile in Pratapgarh. In the latter site, households appeared to suffer more from chronic illnesses, while acute illness episodes were more common in the former. Average self-reported household size varied from 3 in Vaishali to 6 in Kanpur Dehat. A household was usually composed of the head of the house (in the majority of cases the male adult member), his spouse, their children and the parents of the male component. Around 20% of households were headed by women, generally widows.

**Table 1:** **Summary statistics on the household level for the pooled sample and across sites**

**Variables**	**Pooled sample**	**Pratapgarh**	**Kanpur Dehat**	**Vaishali**
Number of households	5215	1542	1751	1922
Household size	4.21(4.37)	4.52(4.45)	6.03(4.77)	3.31(4.00)
Lower wealth third	0.33	0.31(0.46)	0.36(0.48)	0.32(0.47)
Middle wealth third	0.33	0.28(0.45)	0.35(0.48)	0.29(0.45)
Upper wealth third	0.33	0.41(0.49)	0.29(0.45)	0.38(0.49)
Per capita expenditures (in INR)		1128(665)	1793(1653)	1205(947)
Share of health spending on total HH spending	0.21	0.20	0.22	0.20
Number of chronic illnesses (in last 30 days)	0.92 (0.96)	1.29( 1.05)	0.76(0.86)	0.76(0.88)
Number of acute illness episodes (in last 30 days)	1.10(1.06)	1.08(1.11)	1.24(1.12)	0.98(0.95)
Number of hospitalizations (in last 12 months)	0.16(0.42)	0.14(0.38)	0.16(0.43)	0.19(0.44)
Number of pregnancies (in last 12 months)	0.17(0.40)	0.15(0.39)	0.18(0.40)	0.18(0.41)
Proportion of children	0.31(0.23)	0.29(0.21)	0.27(0.22)	0.37(0.23)
Proportion of elderly	0.04(0.12)	0.04(0.12)	0.04(0.13)	0.03(0.11)
Proportion of women at reproductive age	0.27(0.16)	0.31(0.17)	0.27(0.16)	0.28(0.16)
Caste of household head (1/0)				
Scheduled caste/tribe	0.33	0.39	0.31	0.31
Other backward caste	0.56	0.48	0.55	0.63
General caste	0.10	0.12	0.14	0.06
Religion of household head (1/0)				
Hindu	0.90	0.86	0.92	0.92
Muslim	0.10	0.14	0.08	0.07
Other	0.002	0.001	0.002	0.003
Affiliated to a self-help group (1/0)	0.7	0.8	0.6	0.7

Notes: standard deviations between brackets for continuous outcomes.

Table 2 shows descriptive statistics from the retrospective shocks module. Health- related adverse events were the second most common adverse events (34%), after natural disasters (51%). Crop and livestock disease and weddings each affected about 8% of households; all other events were infrequent (and therefore not discussed hereafter)^h^. Adverse health events were equally distributed across socioeconomic status (insignificant CI in Table 2), which is likely to be related to the rather little variation in SES in the sample. This is true for all other events, except for natural disasters that appeared more likely to hit better-off households. The pro-rich concentration of natural disasters might derive from the fact that households need to own sufficient land in order to be affected by a natural disaster. Crop/livestock diseases might be more related to the quality of inputs, and therefore less concentrated with high SES. The fourth and fifth columns of Table 2 show average and expected costs associated with different adverse events. Ill-health and/or deaths costed about 6 times household monthly food spending. Weddings appeared to be most costly^i^, followed by natural disasters, but these switched rankings when considering their expected costs. Even if some discrepancies emerged in the ranking of the different adverse events when considering self-reported costs and severity, the latter confirms that health-related events were perceived as being less severe than weddings (58%) and natural disasters (53%). Only a fourth of households reported to have recovered from illness and/or death in the family (by the time of the survey). Slightly fewer households recovered from natural disasters (21%), and very few households were able to recover from weddings (12%).

**Table 2:** **Descriptive statistics of household shocks**

	**Probability**	**Concentration index**	**Standard error**	**Average cost**	**Expected cost**	**Perceived severe**	**Recovered**
	**Proportion**			**Multiples of monthly food expenditures**		**Proportion**	**Proportion**
Illness or death	0.338	0.003	0.022	5.79	1.955	0.367	0.246
Natural disaster	0.511	0.198	0.042	17.1	8.737	0.534	0.212
Crop, livestock disease	0.076	0.01	0.015	5.445	0.413	0.374	0.304
Job loss/no salary	0.023	−0.025	0.01	7.145	0.163	0.329	0.457
Fall in sale price	0.023	0.011	0.007	6.111	0.142	0.501	0.354
Rise input price	0.013	0.006	0.005	13.833	0.178	0.254	0.205
Conflict	0.013	0.009	0.004	5.167	0.065	0.253	0.084
Wedding	0.076	0.013	0.013	41.203	3.114	0.582	0.12

Notes: Probability of shock occurring, concentration index and standard error, average and expected cost (in multiples of household monthly food spending), perceived severity and recovery.

**Figure 1:**
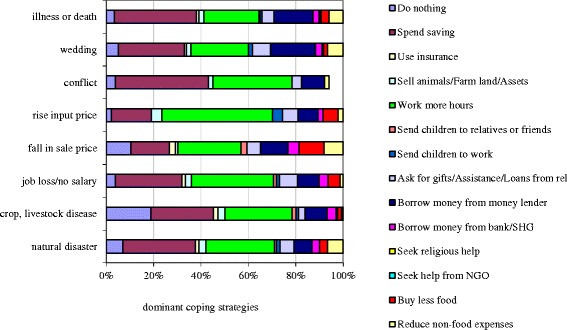
Distribution of most dominant coping strategies for various adverse events.

Generally, spending savings and working more hours were reported as the most relevant coping strategies, followed by borrowing money from a moneylender (Figure 1). Reducing food consumptions was only reported by a minority of households, suggesting that – at least in the short run – most households were able to smooth consumption.

Table 3 provides some further insight into the determinants of the use of the different coping strategies by households hit by adverse events. Disease and/or death and weddings were more likely to lead households to borrow from a moneylender (marginal effects of 0.061 and 0.202 respectively) than natural disasters. Interestingly, the only threat that was more likely than natural disaster to lead to a reduction in food consumption was crop/livestock disease. Results also illustrated that especially poorer households were more likely to work more (7 percentage points), borrow from moneylenders (13 percentage points) and reduce food expenditures (6 percentage points) as compared to those in the upper wealth third.

**Table 3:** **Determinants of coping strategies for various household shocks**

	**Do nothing**	**Spend savings**	**Sell animals/land/assets**	**Work more hours**	**Send children to work**	**Assistance from relatives**	**Borrow from money-lenders**	**Borrow from bank/ SHG**	**Buy less food**	**Reduce non-food expenses**
Proportion elderly	0.06	0.08	0.012	−0.09	−0.028	−0.043	0.06	−0.049	−0.074	−0.033
Proportion children	−0.022	0.077	−0.039	−0.025	−0.064**	0.048	0.164**	−0.016	0.035	−0.009
Proportion reproductive age	−0.052	0.071	−0.006	0.198*	−0.028	0.08	0.06	−0.008	−0.053	−0.104
Scheduled caste/tribe	−0.013	−0.041	−0.003	−0.04	−0.003	0.001	0.026	0.015	−0.041	−0.028
Hindu	0.005	0.029	−0.002	0.021	−0.024	−0.045	0.027	0.036*	0.071	0.059
SHG	−0.013	−0.045*	0.002	−0.02	0.01	0.009	0.005	0.040**	−0.014	−0.003
Pratapgarh	0.092**	0.192**	−0.055**	−0.023	0.004	0.058*	−0.197**	−0.001	−0.108**	−0.133**
Vaishali	−0.128**	0.164**	−0.032*	−0.173**	−0.054**	−0.037	0.324**	−0.003	0.021	0.147**
Middle wealth third	0.045**	0.009	0.024	−0.057	0.001	−0.054**	−0.081**	0.025	−0.025	−0.015
Upper wealth third	0.021	0.105**	0.050**	−0.069*	−0.012	−0.034	−0.131**	0.049**	−0.057*	−0.034
Crop, livestock	0.111**	−0.113**	0.008	−0.07	0.018	−0.024	−0.03	−0.003	−0.145**	−0.02
k disease										
**Illness or death**	**−0.026**	**−0.042**	**0.013**	**−0.049**	**0.014**	**0.028**	0.061*	−0.025*	**−0.014**	**0.025**
Job loss/no salary	−0.009	−0.189**	−0.004	−0.127	0.057	0.037	−0.046	0.096**	0.139*	−0.055
Fall in sale price	−0.015	−0.144*	−0.008	0.027	0.059	0.014	0.003	−0.005	0.233**	−0.243**
Rise input price	−0.034	−0.023	−0.009	0.087	−0.028*	−0.034	0.002	0.021	0.169	−0.232*
Conflict	−0.064**	0.275**	−0.050**	0.279**		−0.092**	−0.214**	−0.051**	0.295**	−0.230*
Wedding	−0.043*	−0.04	0.031	−0.03	0.039*	0.137**	0.202**	−0.005	−0.042	−0.077
Baseline probability	0.120	0.430	0.069	0.551	0.050	0.135	0.322	0.067	0.302	0.486
Observations	5426	5426	5426	5426	5364	5426	5426	5426	5426	5426
Pseudo R2	0.155	0.041	0.035	0.034	0.070	0.032	0.183	0.042	0.093	0.034

Notes: Marginal effects from probit regression. Data on shocks level. Coping strategies are not mutually exclusive. Results are only shown for coping strategies with a baseline probability higher than 0.05. Natural disasters are the omitted shocks category. Standard errors are adjusted for clustering of observations on household level. **significant at 5%, *significant at 10%.

Having described the relative importance of various adverse events threatening household welfare and the main coping strategies employed to deal with these events, the next section provides a more in-depth analysis of the various costs households have to deal with in case of ill-health.

### Household health care-related expenditures

#### Costs composition

Table 4 shows the prevalence and composition of the several types of monthly household health-related expenditures.

**Table 4:** **Distribution of households’ health-related expenditures across the type of care**

	**Proportion of householdsª**	**Proportion of household health spending** ^**a**^	**Direct costs** ^**b**^		**Indirect costs** ^**b**^	
			**Medical fees**	**Medicines and laboratory**	**Transportation and food costs for patient and caregiver**	**Productivity losses for patient and caregiver**
Outpatient care for chronic diseases	49%	43.9%				
Average proportion of total costs (%)			12%	74%	9%	5%
Average spending (INR)			118	1220	159	100
Average spending in (US$)^c^			(2.66)	(27.50)	(3.58)	(2.25)
Standard deviation			257	2825	630	435
Outpatient care for acute conditions	58%	42.7%				
Average proportion of total costs (%)			33%	51%	9%	8%
Average spending (INR)			112	338	67	66
Average spending (in US$)^c^			(2.52)	(7.62)	(1.51)	1.49)
Standard deviation			208	1404	275	237
Hospitalization	15%	8.8%				
Average proportion of total costs (%)			67%	13%	12%	8%
Average spending (INR)			978	212	158	110
Average spending (in US$)^c^			(22.05)	(4.78)	(5.82)	(2.48)
Standard deviation			1208	559	320	229
Maternity care	12%	4.6%				
Average proportion of total costs (%)			15%	2%	61%	23%
Average spending (INR)			56	2	96	42
Average spending (in US$)^c^			(1.26)	(0.05)	(2.16)	(0.95)
Standard deviation			198	9	179	137

Notes: Composition of monthly average household health expenditures (in INR). We include interests that had to be paid on loans taken to finance health care related expenditures in the productivity losses. On average, these represent 8.5% of foregone earnings.

ªamong those households reporting any kind of health expenditures ^b^share of total household health expenditures of the specific type of care ^c^using the 2010 exchange rate.

Among those households that have incurred health expenditures, spending on outpatient care for acute and chronic diseases was quite common (58% and 49% of households respectively), while spending on hospitalization and maternity care was more rare (15% and 12% households respectively). Note that these shares, presented in the first column, add up to 134%, which indicates that quite some households incurred more than one type of health expenditures. In particular, around 30% of households incurred health expenditures for outpatient care for both chronic and acute conditions.

Outpatient care for chronic and acute conditions each took up about 43% of total household health spending, while hospitalizations and maternity care represented about 9% and 5% of the health care budget respectively. Direct costs have been classified into ‘medical fees’ and ‘medicines and laboratory costs’, while indirect costs have been classified into ‘transportation and food costs (for patient and caregiver)’ and ‘productivity losses (based on self-reported information) for the patient and caregiver’. The bulk of expenditures on care for chronic diseases (74%) were related to additional medical services, mostly drugs. Also for outpatient spending on acute conditions about half of the costs were related to drugs and tests, while only a third was spent on doctor fees. Concerning hospitalizations, the medical fees were much more important (67%), while medicines and test costs represented 13% of total costs. The shares of indirect costs (transportation and food, as well as the loss of productivity) were highest for maternity care (61% and 23% respectively), which is likely to be related to the relatively low user fees associated with maternity care (mostly for free in public facilities). The shares of non-medical costs were about 10% each for outpatient care and hospitalizations. Loss of productivity represented a rather small proportion of the costs associated with outpatient care for chronic (5%) and acute (8%) conditions and for hospitalizations (8%).

We have also investigated heterogeneity of results across the types of ill-health conditions. Reported symptoms of both acute and chronic conditions were categorized into communicable and non-communicable diseases (NCDs) using ICD10 codes developed by the World Health Organization [17]. Among the 63% of illness episodes which we were able to classify we found a higher prevalence of non-communicable diseases (85%) than communicable diseases (15%). Households’ average monthly costs related to non-communicable diseases (1573 INR) were higher than those related to communicable diseases (1261 INR). In particular, additional medical costs for NCDs were the largest cost component (923 INR per household per month). These results might suggest a growing (economic) burden of NCDs in rural India.

#### Coping with health expenditures

**Figure 2:**
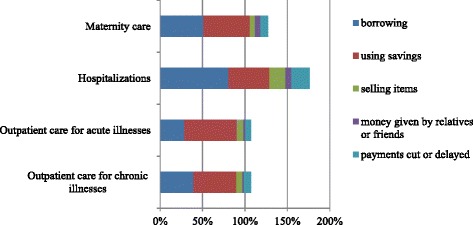
Relative importance of coping strategies for financing health-related expenditures by type of care. Notes: Bars represent the proportion of households confronted with a specific type of health care use that have used a specific coping strategy. Households can employ more than one coping strategy.

Having established the various costs associated with ill-health, we now want to investigate how households finance these costs in order to better understand the potential long-term consequences. Figure 2 shows the relative importance of different coping strategies for different types of care and reveals that, particularly for hospitalizations, households resort to a combination of multiple financing mechanisms. Remarkably, over 80% of households that have been confronted with a hospitalization in the past year have borrowed money to cope with these expenditures. Other types of health care expenditures were typically financed through savings and loans, and to a lesser extent by selling assets and cutting/delaying payments. Those households that did sell assets mostly sold agricultural equipment and grain (58% of total cases), followed by household items (16%), livestock (11%) and jewelry (9%). Households that reported to cut back on spending mostly did this for food-related spending (68%).

Confirming earlier results, we find that cutting or delaying payments was only reported by a minority of households and most often in relation to hospitalizations (21.10%). Comparing average amounts obtained through each of the coping strategies revealed that selling assets on average contributed 1298 INR, followed by borrowing and delaying payments (both 1064 INR), receiving money (962 INR) and using savings (533 INR).

Table 5 explores the factors correlated with various financing mechanisms – much like in Table 3 but with data shaped on illness level rather than on shocks level. Households appeared more likely to need alternative financing sources for inpatient care, which is probably related to the fact that hospitalizations come quite unexpected and are generally more expensive. A hospitalization increased the probability of having to borrow by 0.36 percentage points, which is a dramatic effect, considering the baseline probability of 0.39. Hospitalizations were also more likely to lead to a reduction in consumption (marginal effect of 0.1). As expected, wealthier households were more likely to use savings and less likely to borrow money to finance health-related costs, while households affiliated to SHGs were slightly more likely to borrow money, probably due to their easier access to credit from the micro-credit network. The geographical indicators (Pratapgahr and Vaishali) remained very significant, even after controlling for all other covariates, indicating that there were indeed substantial differences in the ways people cope with health care expenditures across locations. Households in Pratapgarh were, for example, more likely to rely on their savings and on the support of their relatives for covering health-related expenditures and less likely to borrow from moneylenders, sell assets or reduce food and non-food expenditures. This is likely to be related to their higher SES.

**Table 5:** **Determinants of coping strategies for various types of healthcare**

**Variable**	**Borrowing**	**Spend savings**	**Selling items**	**Money from friends and relatives**	**Cutting back on spending or delaying payments**
Outpatient chronic	0.013	−0.001	0.005	−0.029**	0.030
Outpatient acute	−0.166***	0.061*	−0.013	−0.022*	−0.013
Inpatient	0.360***	−0.050	0.078***	0.012	0.100***
Proportion elderly	−0.153	−0.002	−0.002	0.015	0.009
Proportion children	−0.018	−0.007	−0.002	0.035	0.037
Proportion reproductive age	0.068	0.010	−0.031	−0.023	0.045
Scheduled caste/tribe	0.015	−0.035	−0.022	0.008	−0.006
Hindu	−0.019	0.012	0.006	0.003	−0.000
SHG	0.038**	−0.020	0.000	−0.003	0.012
Pratapgarh	−0.129***	0.252***	−0.152***	−0.007	−0.092***
Vaishali	−0.013	0.118***	−0.179***	0.029***	0.021
Middle wealth third	−0.056*	0.040	0.028	0.028**	−0.014
Upper wealth third	−0.190***	0.123***	0.016	0.026**	−0.001
Baseline probability	0.39	0.59	0.09	0.03	0.09
Observations	8540	8540	8540	8540	8540
Pseudo R2	0.0949	0.0449	0.1391	0.0820	0.0592

Notes: Marginal effects from probit regression. Data on illness level. Coping strategies are not mutually exclusive. Maternity care is the omitted healthcare use category. Standard errors are adjusted for clustering of observations on household level. ***significant at 1%, **significant at 5%, *significant at 10%.

The welfare implications of borrowing to finance health expenditures depend to a large extent on the interest that has to be paid back, typically correlated with the type of lender. The average interest rate (on a monthly basis) among all loans taken is 3%^j^. Borrowing from moneylenders was at an average interest rate of 5% while SHGs only charged around 2% per month. In our data households mostly borrowed money from friends or neighbors (41%) and from moneylenders (26%).

Using probit models for the choice of lender (presented in Table 6), we also found that hospitalizations were more likely to push households to borrow from moneylenders (as compared to maternity and outpatient care). Households affiliated to SHGs were much more likely to use the SHG informal credit system to finance health costs. However, the saved amount of a SHG was usually not so capacious to cover repeated or very high health expenditures, which is why many SHG members have, nonetheless, often needed to recur to other financial sources.

**Table 6:** **Determinants of HHs’ borrowing behavior**

**Variable**	**Relatives**	**Friends, neighbours**	**SHGs**	**Moneylender**	**Doctor or hospital**
Outpatient_chronic	−0.049*	0.002	0.012	0.045*	−0.006
Outpatient_acute	−0.079***	0.095***	−0.016	−0.029	0.026*
Inpatient	0.065**	−0.177***	0.006	0.124***	−0.122***
Proportion elderly	−0.122	0.193	0.076	−0.113	−0.074
Proportion children	0.029	−0.076	0.012	−0.017	0.037
Proportion reproductive	0.040	−0.075	0.024	−0.065	0.091**
Scheduled	−0.004	−0.043	−0.017*	0.032	0.025*
Hindu	−0.034	0.035	0.016	−0.025	−0.027
SHG	−0.031	−0.047*	0.077***	0.038*	−0.009
Kanpur	0.141***	0.260***	0.025*	−0.302***	−0.055***
Allahabad	0.088***	0.291***	0.070***	−0.417***	−0.017
Medium wealth third	−0.001	0.006	−0.009	−0.002	0.010
Upper wealth third	0.018	0.027	0.005	−0.055*	0.018
Observations	3406	3406	3406	3406	3406
Baseline probability	0.18	0.41	0.06	0.26	0.05
Pseudo R2	0.1469	0.1404	0.0956	0.1546	0.0309

Notes: Marginal effects from probit regressions on each of the main borrowing types. Data is at illness level. Regressions are only run on the sample of illnesses for which money was borrowed. Models account for clustering of observations on the household level and sample weights. Maternity care is the omitted healthcare use category. ***significant at 1%, **significant at 5%, *significant at 10%.

#### Foregone care

Finally, it should be noted that our analysis has not considered those households that were not able to cover health expenditures and therefore decided to forego using health care. While foregoing health care saves health care costs in the short run, it can lead to very severe health and productivity/income losses in the long run [13]. In our data 18% of respondents reported to have foregone care (when needed) at least once in the 30 days preceding the survey. This is likely to be an underestimation, given that there may be a lot of unperceived need in this context. Most of the episodes of foregone care were related to chronic conditions (54%) and acute episodes of illness (34%). The main (reported) reasons for not seeking healthcare were the high costs of medical care (52.4%) and the inaccessible price of drugs and medical tests (35.9%).

## Discussion

As compared to other countries in South East Asia, India’s health care system is characterized by very high out of pocket payments, and consequently low financial protection and access to care [3,7,11]. In this context, ill-health can pose severe economic threats to households, many of which already suffer from economic hardship.

This paper shows that ill-health is the second most common threat to households’ welfare in rural Uttar Pradesh and Bihar, next to natural disasters, but while the latter are more likely to hit richer households, health related shocks are more equally distributed across socioeconomic status. The high prevalence of health shocks emerged in our study is in line with the results from other developing countries [16,22-25].

Households employ a wide variety of coping strategies, but only a quarter of them report to have been able to recover from the health-related expenditures. Interestingly, weddings are the most costly events for households, but these obviously do not come unexpected.

Our analysis also highlights the importance of expenditures on chronic conditions and non-communicable diseases (NCDs). Close to half of households’ health expenditures are made for chronic conditions, and 74% of these are made on drugs. The ‘chronic emergency’ in the developing world is increasingly recognized, with NCDs expected to account for two-thirds of the disease burden in 2030 in middle-income countries [26] and to cause yearly economic losses in the magnitude of 4% of these countries’ Gross Domestic Products (GDP) [27]. Mahal et al. [28] use Indian national data for the year 2004 and estimate that India’s GDP would have been 4-10% higher without the existence of NCDs. Our findings on households’ health expenditures are consistent with those of Dror et al. [7], studying healthcare costs in five resource poor locations in rural India and finding a ratio of direct to indirect cost of illness of 67:30 (compared to our 66:34). Dror et al. also confirm our findings concerning a high prevalence of costs for outpatient care, with acute illnesses representing 37.4% of total costs, followed by 32% for chronic illnesses, while hospitalizations represented only 11% of total costs.

Loss of productivity represents the smallest costs component for our target communities, indicating that households are able to secure household income when confronted with ill-health, at least in the short term. It should be noted that loss of productivity did not take into account the welfare losses of women not being able to perform domestic duties. Rugalema [29] found that indirect costs related to women are higher than those for men within the same household. Furthermore, given the difficulties for respondents in estimating income losses (especially for agricultural production), it is possible that these are underestimated in our data.

Households use a variety of strategies to cope with health-related expenditures, especially in the case of hospitalizations. The most important coping strategies are using savings, selling assets and borrowing, all of which entail important long-term consequences for households’ welfare. Selling productive assets represents one of the most corrosive coping strategy in developing countries, as it compromises the ability to generate income in the future [13,24]. Moneylenders can offer seemingly attractive long-term financing with frequent payment of interest, leaving the borrowers unable to repay the principal amount borrowed^k^. Furthermore, the loan is often combined with mortgage on land or other properties. Our findings are similar to those obtained by Binnendijk et al. [11] from rural communities in Orissa, India. Their study also reports a high prevalence of using savings and borrowing money (especially for coping with hospitalization costs) as coping strategies.

Few households report having to reduce (food) consumption in response to ill-health, suggesting that - at least in the short run - households are able to smooth consumption in the event of ill-health.

There are some limitations to our analysis. Most importantly, the cross-sectional nature of the data does not allow deriving any causal relations. Second, much of the analysis on shocks and coping strategies rely on self-reported data which might be prone to reporting bias. Third, since our data are collected within a rather specific (and homogenous) population, there are some limits to the generalizability of our results.

## Conclusions

This paper concludes that ill-health poses an important economic threat to relatively poor households in rural northern India and that, while households seem to be able to find ways to finance health-related costs in the short term, there are important long term implications for households’ welfare. Furthermore, a substantial share of households forwent seeking healthcare, which has severe consequences on the productivity capacity and on the health capital of community members in the long term. The emerged importance of expenditures on chronic conditions suggests that ‘health shocks’ should not only be thought of in terms of acute unexpected illness episodes, but also in terms of the onset of a chronic disease which requires (expected) spending over a long period of time. Retrospective survey tools like the one presented in this paper might therefore not get complete information on the way ill-health threatens households economically.

As most of the economic risk from ill-health appears to be related to OOP spending, introducing health insurance, that pre-finances these expenditures and pools risks within the community, may contribute significantly to alleviate economic hardship for families in rural India. The importance of care for chronic diseases, however, represents a big challenge for the sustainability of community-based health insurance schemes, since it is necessary to ensure a sufficient degree of risk pooling.

## Endnotes

^a^Tesliuc and Lindert [22]; Kenjiro [23]; Dercon and Hoddinott [24]; Heltberg and Lund [25]; Wagstaff and Lindelow [16] provide evidence for other countries. Ill-health appears to be one (in some cases the most) prevalent and costly shock in the studied countries, respectively Guatemala, Cambodia, Ethiopia, Pakistan and Laos.

^b^More information on the project and the procedure of random sampling can be found in Doyle et al. [30].

^c^Some SHGs grouped themselves into SHGs Federations, which are formal institutions (registered as societies) and show several benefits, such as strong political influence, development of economies of scale and access to greater capital [31,32].

^d^Bihar and Uttar Pradesh are amongst India’s most populated, poorest and least urbanized states, and in so far as SHG households are typically poorer and less educated than the general population, our analysis focuses on a relatively marginalized population in rural India [33].

^e^The Indian Council of Medical Research issued the “Ethical guidelines for biomedical research on human participants” in 2006. The document is available at: http://icmr.nic.in/ethical_guidelines.pdf (accessed 24.02.2015).

^f^Concerning the financing mechanisms, it must be noted that –unfortunately –the retrospective shocks tool and the health care survey section differ quite substantially in terms of the sequencing and alternative coping responses provided and, perhaps most importantly, the type of health events concerned (the shocks section includes deaths within the household among the health shocks).

^g^Recently, Erreygers [21] has shown that the standard concentration index, when applied to bounded indicators (such as binary variables) does not satisfy the mirror condition and suggested a correction.

^h^To investigate the idiosyncrasy of events, linear regressions were estimated of the specific shock indicator on a set of village dummies. In general, all shocks appear quite idiosyncratic, with village effects never explaining more than 7% of the variation. Natural disasters are typically more concentrated within villages.

^i^Bloch and Rao [34] find that dowries amount to six times average incomes among pottery families in South Karnataka.

^j^Households were asked “On every 100 rupees you borrowed, how many extra rupees do you pay back?”.

^k^We do not possess detailed information on the time needed by households to repay the loan. On average people report repayments amounting to 7.6% of the amount borrowed. However, considering that the monthly interest rate amounted to 2 to 5% and that most of the people reported being able to make repayments only for “what they can, when they can” (65%) or by supply of labor (16%), we can assume that loans are not quickly paid back.

## Additional file

Additional file 1:
**Retrospective household shocks module.**
