# Supplementary material for: Economic consequences of ill-health for households in northern rural India
Source: BMC Health Serv Res. 2015 Apr 26;15:179. doi: 10.1186/s12913-015-0833-0 (PMC4419476; doi:10.1186/s12913-015-0833-0)
Supplement: Additional file 1: — Retrospective household shocks module. [file 12913_2015_833_MOESM1_ESM.pdf]

10.6 COPING WITH FALLS IN INCOME AND LARGE EXPENSES, LAST 12 MONTHS							
[Read Out:] *I want to ask you about a list of adverse events that your household might have experienced, and how you coped with them.*							
	10.6.1 How many times did this event happen in the last 12 months? *If never, write 0*	10.6.2 In the last 12 months, how severely did this event affect your household? *Code*	10.6.3 How much has your household recovered from the consequences of this event? *Code*	10.6.4 What was the total value lost due to this event regarding income, expenditures and assets (incl land)? *Rs.*	10.6.5 In order, what were the three most important things you did to cope with the financial problems this event caused? *Code*		
					1st.	2nd.	3rd.
A. Natural Disaster (Drought / Flood / etc.)							
B. Storage, Crop or Livestock disease / pest							
C. Job Loss / No salary							
D. Drop in sale prices of agricultural produce							
E. Increase in agricultu ral input prices							
F. Communal / Political / Religious / Tribal conflict/ Crime							
G. Wedding							
H. Illness or death of household member							

Code box for Question 10.6.2

No Impact....1
Low.........2
Medium......3
High........4
Extreme.....5

Code box for Question 10.6.3

Completely....1
Mostly........2
Somewhat......3
A little......4
Not at all....5

Code box for Question 10.6.5

Do nothing....1
Spend savings....2
Use insurance....3
Sell animals/ farm land/ assets....4
Work more hours....5
Send children to relatives or friends....6
Send children to work....7
Ask for gifts/ assistance/ loans from relatives and friends....8
Borrow money from money lender....9
Borrow money from Bank /SHG....10
Seek religious / spiritual help....11
Get help from NGO....12
Buy less food....13
Reduce non-food expenses....14
Other....98 *(please specify)*

Appendix 1: Retrospective household shocks module included in the survey.
